# Classification Accuracies of Physical Activities Using Smartphone Motion Sensors

**DOI:** 10.2196/jmir.2208

**Published:** 2012-10-05

**Authors:** Wanmin Wu, Sanjoy Dasgupta, Ernesto E Ramirez, Carlyn Peterson, Gregory J Norman

**Affiliations:** ^1^Center For Wireless & Population Health SystemsDepartment of Family & Preventive MedicineUniversity of California, San DiegoLa Jolla, CAUnited States; ^2^Department of Computer Science and EngineeringUniversity of California, San DiegoLa Jolla, CAUnited States

**Keywords:** Activity classification, machine learning, accelerometer, gyroscope, smartphone

## Abstract

**Background:**

Over the past few years, the world has witnessed an unprecedented growth in smartphone use. With sensors such as accelerometers and gyroscopes on board, smartphones have the potential to enhance our understanding of health behavior, in particular physical activity or the lack thereof. However, reliable and valid activity measurement using only a smartphone in situ has not been realized.

**Objective:**

To examine the validity of the iPod Touch (Apple, Inc.) and particularly to understand the value of using gyroscopes for classifying types of physical activity, with the goal of creating a measurement and feedback system that easily integrates into individuals’ daily living.

**Methods:**

We collected accelerometer and gyroscope data for 16 participants on 13 activities with an iPod Touch, a device that has essentially the same sensors and computing platform as an iPhone. The 13 activities were sitting, walking, jogging, and going upstairs and downstairs at different paces. We extracted time and frequency features, including mean and variance of acceleration and gyroscope on each axis, vector magnitude of acceleration, and fast Fourier transform magnitude for each axis of acceleration. Different classifiers were compared using the Waikato Environment for Knowledge Analysis (WEKA) toolkit, including C4.5 (J48) decision tree, multilayer perception, naive Bayes, logistic, k-nearest neighbor (kNN), and meta-algorithms such as boosting and bagging. The 10-fold cross-validation protocol was used.

**Results:**

Overall, the kNN classifier achieved the best accuracies: 52.3%–79.4% for up and down stair walking, 91.7% for jogging, 90.1%–94.1% for walking on a level ground, and 100% for sitting. A 2-second sliding window size with a 1-second overlap worked the best. Adding gyroscope measurements proved to be more beneficial than relying solely on accelerometer readings for all activities (with improvement ranging from 3.1% to 13.4%).

**Conclusions:**

Common categories of physical activity and sedentary behavior (walking, jogging, and sitting) can be recognized with high accuracies using both the accelerometer and gyroscope onboard the iPod touch or iPhone. This suggests the potential of developing just-in-time classification and feedback tools on smartphones.

## Introduction

It is widely recognized that lack of physical activity and excess of sedentary behavior are associated with increased health risks for obesity, type 2 diabetes, cardiovascular disease, depression, and all-cause mortality [1]. In the United States, physical inactivity is alarmingly prevalent. A study based on the 2003-2004 National Health and Nutrition Examination Survey data suggested that on average Americans spend about 55% of their waking time, or 7.7 hours per day [2], being sedentary. Another study based on the same data showed that less than 5% of adults meet the national 30 minutes/day guideline for physical activity [3].

Measurement of physical activity and sedentary behavior is a fundamental, yet nontrivial, task for developing effective intervention tools. Self-reported data are subject to bias and errors. Objective methods enabled by advancement in accelerometer technologies are gaining increasing attention. Researchers have explored different accelerometer-equipped monitoring devices, such as customized sensor boards [4,5], Actigraph accelerometer [6,7], DynaPort [8], and Pegasus activity monitors [9], for detecting activities. Although some of these devices are small, they are still an extra burden for users to wear.

More recently, smartphones equipped with accelerometers have become ubiquitous. Carried by people throughout the day, smartphones are an ideal platform for monitoring physical activity and sedentary behavior and for just-in-time intervention. Furthermore, they have powerful computational capabilities and allow development of customized applications that integrate monitoring and intervention. Researchers have used accelerometers on Nokia N95 phones [10,11] and Android phones [12] to detect common activities such walking, stair climbing, jogging, and sitting.

Similar to earlier studies [10-12], in this study the goal was to create a valid activity classification tool that uses sensors onboard today’s smartphones. However, this study is distinguished by the following three characteristics.

### Device

We conducted our study with the iPod Touch (Apple Inc., Cupertino, CA, USA). It has essentially the same sensors and computing platform as the iPhone, yet costs much less. We compared the obtained results with findings from the two previous studies that used the Nokia N95 and Android phones.

### Sensor

While past work relied mainly on accelerometers on cell phones, we combined acceleration with orientation readings from the newly available gyroscope sensor. In June 2010, Apple became the first to introduce gyroscopes to a mobile phone with the launch of the iPhone 4. Since then, an increasing number of mobile phones have added gyroscopes on board. However, few researchers have explored the use of gyroscopes as a way to measure physical activity. This study demonstrated one of the first steps in assessing whether gyroscope readings are beneficial in classifying activities. As accelerometers measure acceleration, gyroscopes measure rotation. Our hypothesis was that combining these two complementary sensors could improve recognition accuracy of activities.

### Activity Intensity

Previous research [10-12] classified common physical activities such as walking on stairs and walking on level ground without differentiating speed. However, it has been shown that the intensity of these activities matters: walking at a normal pace is classified as light physical activity with an intensity of <3 metabolic equivalents (work metabolic rate/resting metabolic rate), whereas brisk walking is considered moderate physical activity with an intensity of 3-6 metabolic equivalents [13]. The US Centers for Disease Control and Prevention guideline on physical activity for adults is 150 minutes of moderate-intensity aerobic activity (eg, brisk walking) every week. Thus, we differentiated the speed of common activities, such as walking and stair climbing, at normal and brisk paces.

## Methods

### Data Collection

#### Recruitment

Eligibility criteria were being 19–60 years of age, speaking English, having no existing medical conditions that prevent performing moderate-intensity physical activity, and being able to climb and descend stairs. We drew a convenience sample from the University of California, San Diego. Men were recruited through the university’s campuswide listserv. About 43 men responded with interest in participating and 6 were recruited. We recruited 5 women within the Center for Wireless & Population Health Systems in the California Institute for Telecommunications and Information Technology and 5 from a pool of potential study participants at the Moores Cancer Center, San Diego, CA, USA. All participants signed study consent forms and all protocols were approved by the university’s institutional review board. Table 1 shows the characteristics of participants.

#### Hardware Platform

**Figure 1:**
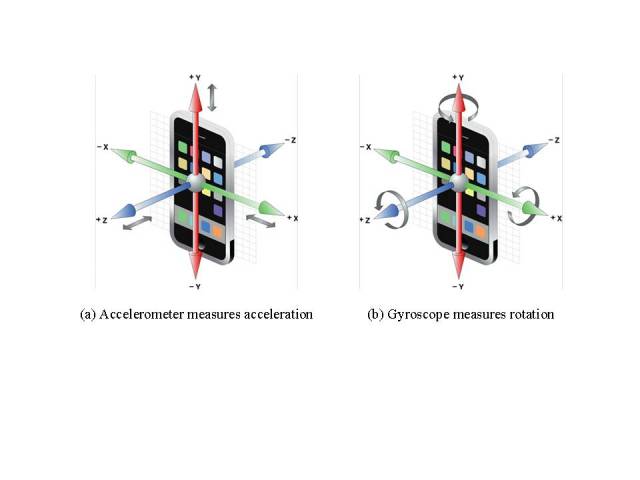
Coordinate system of the iPod touch (figure courtesy of Apple Inc.).

We used the iPod Touch (Apple Inc.) as the hardware platform for data collection. It has essentially the same accelerometer and gyroscope sensors as the iPhone (Apple Inc.) (Figure 1), one of the most widely used smartphones on the market. They run the same iOS operating system as well. The fourth-generation iPod Touch we employed used STMicroelectronics (Geneva, Switzerland) LIS331DLH accelerometer and L3G4200D gyroscope [14]. The iPod Touch is 11.18 cm high, 5.89 cm wide, and 0.71 cm deep, and weighs 100.9 g.

**Table 1:** Demographic characteristics of study participants (n = 16).

Characteristic		n	%
**Age group (years)**			
	21–30	6	38%
	31–40	3	19%
	41–50	1	6%
	51–60	6	38%
**Gender**			
	Female	10	63%
	Male	6	38%
**Body mass index range (kg/m^2^)**			
	18.5–24.9 (normal)	8	50%
	25–29.9 (overweight)	1	6%
	30–34.9 (moderately obese)	4	25%
	35–40 (severely obese)	3	19%

#### Software Setup

We developed an application on the iPod Touch for easy data collection. It allows users to specify an activity type and the device location (often referred to as the labeling or annotation step), start and stop data recording using a toggle button, and transmit collected data to research staff. We used the CMDeviceMotion class of iOS 4.2 application programming interface that encapsulates processed acceleration and gyroscope measurements. More specifically, we used the userAcceleration property of CMDeviceMotion to get the 3-axis acceleration (gravitational force) that the user imparts to the device (ie, total acceleration minus gravity), and the rotationRate property to get the device’s rate of rotation (in radians per second) around three axes, with the gyroscope bias removed by Apple’s proprietary Core Motion algorithms. Both accelerometer and gyroscope were configured to sample at a 30 Hz (33.33 milliseconds) rate.

#### Activities

We studied 13 activity types in total, 4 of which were paced by research staff in a laboratory setting on a treadmill, and the rest were self-paced by participants to simulate a free-living condition. The details of these activities are described in Table 2. As shown in the last column, these 13 activities were further grouped into 9 classes: slow walking, normal walking, brisk walking, jogging, sitting, normal upstairs, normal downstairs, brisk upstairs, and brisk downstairs. For example, the prescribed laboratory activity A2 (3.0 mph walking) and the self-paced activity A11 (400 m normal walking) belong to the same class, C2 (normal walking). That is, a classification system should be able to recognize both activities as normal walking.

**Table 2:** Physical activity type descriptions and classifications.

Activity name		Description	Class
**Prescribed**			
	A1. 1.5 mph walking	Walking at 1.5 mph on a treadmill for 3 minutes	C1. Slow walking
	A2. 3.0 mph walking	Walking at 3.0 mph on a treadmill for 3 minutes	C2. Normal walking
	A3. 4.0 mph walking	Walking at 4.0 mph on a treadmill for 3 minutes	C3. Brisk walking
	A4. 5.5 mph jogging	Jogging at 5.5 mph on a treadmill for 3 minutes	C4. Jogging
**Self-paced**			
	A5. Sitting	Seated in a chair, remaining still	C5. Sitting
	A6. Normal upstairs	Ascending one flight of stairs (19 steps) at a normal pace	C6. Normal upstairs
	A7. Normal downstairs	Descending one flight of stairs (19 steps) at a normal pace	C7. Normal downstairs
	A8. Brisk upstairs	Ascending one flight of stairs (19 steps) at a brisk pace	C8. Brisk upstairs
	A9. Brisk downstairs	Descending one flight of stairs (19 steps) at a brisk pace	C9. Brisk downstairs
	A10. 400 m slow walking	Walking for one lap around a 400 m track at a slow pace	C1. Slow walking
	A11. 400 m normal walking	Walking for one lap around a 400 m track at a normal pace	C2. Normal walking
	A12. 400 m brisk walking	Walking for one lap around a 400 m track at a brisk pace	C3. Brisk walking
	A13. 400 m jogging	Jogging for one lap around a 400 m track.	C4. Jogging

#### Collection Protocol

Due to limited treadmill and track availability, not every participant completed the activities in the order specified in Table 2. Some participants followed that order, while others completed the 400 m track activities (A10–A13) first, followed by stair tasks (A6–A9), and finally the laboratory activities (A1–A5). The participant carried the iPod touch device in an armband for jogging and in a front shorts pocket for all other activities. When in the pocket, the device was oriented with the screen facing away from the body and the 30-pin connection port facing up. When the device was in the armband, the screen was faced away from the body with the 30-pin connection port facing down. Not all participants were able to complete all tasks. For example, approximately half of the participants were unable to perform all of the strenuous jogging activities (A4. 5.5 mph jog and A13. 400 m jog). The whole protocol took about 2 hours to complete. Each participant received US $50 as compensation.

### Data Preprocessing

The time series of collected sensory data (30 Hz) were stored in a comma-separated values file per activity, per participant. The beginning and the end of all files were manually trimmed in a data preprocessing phase. This was because at the beginning of data recording the research staff had to start the data recording by pressing a toggle button and then place the device in the right position on the participant; at the end of the activity they collected the device and stopped data recording.

### Feature Extraction

#### Window Size

The data vector containing 3-axis acceleration and 3-axis rotation rate recorded at a time instant is called a *sample*. To reduce noise and capture cyclic patterns of motion, features were not computed on each single sample, but on a sliding window of samples. Many studies have indicated the superiority of using a 1-second window size [4,11,15,16]; others have used larger window sizes such as 2 seconds [9] and 10 seconds [12] to capture more cyclic patterns. We experimentally compared window sizes of 1 second, 2 seconds, 5 seconds, and 10 seconds, and found that the 2-second window size (60 samples in our case) produced the best classification performance. The detailed comparison results are shown in the next section. Finally, the use of a 50% overlap between consecutive windows has been shown to be beneficial by past research [17]. Thus, we used a 1-second overlap in our sliding windows.

#### Features

As discussed before, we used an accelerometer and gyroscope as our signal sources. In the literature, different features have been employed for acceleration-based activity recognition, such as mean [4,5,9,12], variance [11,12], spectral entropy [4, 5], and fast Fourier transform coefficients [5,9,11,18]. To select features, we performed extensive comparative experiments on these various features. The following features (variants of 4 basic ones) produced the best classification results:

- *Mean* for each axis of acceleration, each axis of rotation rate (from gyroscope), and acceleration magnitude, computed as the square root of (*A^2^_x_ + A^2^_y_ + A^2^_z_*) in the sliding window
- *Standard deviation* for each axis of acceleration, each axis of rotation rate, and acceleration magnitude in the sliding window
- *Sum* of acceleration magnitude in the sliding window
- *Fast Fourier transform magnitude*, or magnitude of the first five coefficients of the fast Fourier transform power spectrum (for each axis of acceleration). As Preece et al [9] showed, this fast Fourier transform feature is overall the best-performing feature among all compared time, frequency, and wavelet features for all activities.

## Results

### Classifier Comparison

We used the Waikato Environment for Knowledge Analysis (WEKA) machine learning toolkit [19] to train and compare the performance of the classifiers C4.5 (J48) decision tree, multilayer perception, naive Bayes, logistic, and k-nearest neighbor (kNN). For all classifiers, the default WEKA settings (version 3-6-6) were used. We used 10-fold cross-validation for all experiments. Table 3 shows the comparison results using a 2-second window size. Among these basic classifiers, kNN generally produced the best accuracy results. Thus, we further applied meta-algorithms including boosting (AdaBoostM1) and bagging to the kNN classifier but observed no clear benefits as shown in Table 3.

**Table 3:** Comparison of classification accuracies by classifier.

Activity	kNN^a^	J48^b^	MLP^c^	Logistic	NB^d^	Boosting	Bagging
C1. Slow walking	94.1%	86.3%	90.8%	88.3%	61.3%	94.1%	94.1%
C2. Normal walking	92%	80.9%	84.6%	74.2%	55.7%	92%	92.2%
C3. Brisk Walking	90.1%	82.2%	85%	68.7%	64.9%	89.9%	90.1%
C4. Jogging	91.7%	91.7%	91.5%	92.2%	79%	92.2%	91.7%
C5. Sitting	100%	99.6%	100%	100%	98.5%	100%	100%
C6. Normal upstairs	69.8%	51%	42.7%	47.9%	30.2%	69.8%	69.8%
C7. Normal downstairs	79.4%	64.9%	54.6%	46.4%	32%	79.4%	77.3%
C8. Brisk upstairs	70.4%	69%	33.8%	19.7%	22.5%	70.4%	69%
C9. Brisk downstairs	52.3%	44.6%	24.6%	33.8%	35.4%	52.3%	43.1%
Weighted average	90.2%	83.0%	83.4%	77.2%	63.2%	90.2%	89.9%

^a ^k-nearest neighbor.

^b ^C4.5 decision tree.

^c ^Multilayer perception.

^d ^Naive Bayes.

In general, the kNN classifier achieved high accuracies for walking at different paces (90.1%–94.1%), jogging (91.7%), and sitting (100%). Stair walking proved to be the most challenging activity, with recognition accuracies ranging from 52.3% to 79.4%.

Table 4 presents the confusion matrix generated by kNN. Among all the misclassified sample segments (n = 274), a significant number were caused by the difficulty of differentiating walking at different speeds (n = 101) and differentiating walking on stairs from walking on a level ground (n = 103). Fortunately, compared with walking on level ground (which has a classification accuracy of 90.1%–94.1%), stair walking is only a small part of daily activities for most people. Confusion also existed between brisk walking and jogging (with n = 57 sample segments incorrectly classified). This may be due to the different speeds participants used in self-paced situations. It is interesting to focus on the spectrum from slow walking to jogging. These activities were almost never confused with staircase motion or with sitting. Moreover, the predicted activity was almost always either correct or one speed gradation off; for instance, slow walking was never mistaken for jogging.

**Table 4:** Confusion matrix (k-nearest neighbor classifier with accelerometer and gyroscope features).

Activity	Classified as...								
	C1	C2	C3	C4	C5	C6	C7	C8	C9
C1 = Slow walking	572	30	5	0	0	0	0	0	0
C2 = Normal walking	29	602	13	0	0	4	5	0	1
C3 = Brisk walking	7	17	475	25	0	0	1	0	2
C4 = Jogging	0	1	32	389	0	0	0	2	0
C5 = Sitting	0	0	0	0	266	0	0	0	0
C6 = Normal upstairs	8	15	2	0	0	67	4	0	0
C7 = Normal downstairs	6	7	3	0	0	4	77	0	0
C8 = Brisk upstairs	1	8	10	1	0	0	0	50	1
C9 = Brisk downstairs	0	16	14	0	0	1	0	0	34

### Window Size Comparison

As discussed above, different window sizes have been used in the literature, including 1 second [3,9,15], 2 seconds [20], and 10 seconds [11]. We experimentally compared window sizes of 1 second, 2 seconds, 5 seconds, and 10 seconds using the kNN classifier, with overall accuracies of 87.7%, 90.2%, 88.5%, and 84.2%, respectively. The 2-second window size achieved the best overall classification performance in terms of weighted average accuracy.

### Effect of Gyroscope

We made one of the first attempts to evaluate the effect of a gyroscope in measuring physical activities. Our hypothesis was that adding gyroscope data could improve the overall classification accuracy. This was confirmed by the results as shown in Table 5. Using both rotation rate (from the gyroscope) and acceleration features (from the accelerometer) with kNN resulted in higher accuracies for all activity classes than when using only acceleration features, with improvement ranging from 3.1% to 13.4%.

Gyroscope data are useful because almost all activities involve some sort of orientation change of the phone. This makes it a powerful complementary data source to the accelerometer, which only measures linear motion along specified directions.

**Table 5:** A comparison of classification accuracies using acceleration features only versus using both acceleration and rotation rate features (k-nearest neighbor classifier).

Activity	Acceleration	Acceleration+ rotation rate	Difference
C1. Slow walking	89.6%	94.1%	+4.5%
C2. Normal walking	85.8%	92%	+6.2%
C3. Brisk walking	78%	90.1%	+12.1%
C4. Jogging	85.4%	91.7%	+6.3%
C5. Sitting	100%	100%	0%
C6. Normal upstairs	65.6%	69.8%	+4.2%
C7. Normal downstairs	66%	79.4%	+13.4%
C8. Brisk upstairs	64.8%	70.4%	+5.6%
C9. Brisk downstairs	49.2%	52.3%	+3.1%
Weighted average	83.7%	90.2%	+6.5%

### Comparison With Prior Work

Table 6 shows a comparison of classification accuracies obtained in our study against those reported in three previous studies. However, the differences between studies should be interpreted with caution because they can be attributed to many factors (as listed in Table 7). The most significant factor is that different datasets were used in each study.

The lack of a shared dataset in the research community makes cross-study comparison difficult, particularly on feature types, sliding window sizes, and classifiers. To accelerate future research on assessment of activity using smartphones, we are sharing our anonymized iPod touch dataset with the research community. The dataset is accessible through the iDash Data Repository [20].

**Table 6:** Accuracy comparison with prior work.

Activity	Lu et al [10]	Reddy et al [11]	Kwapisz et al [12]	Current study
Sitting (still)	97.7%	95.6%	95%	100%
Walking	96.6%	96.8%	91.7%	94.1% (slow), 92% (normal), 90.1% (brisk)
Running or jogging	98%	91.0%	98.3%	91.7%
Upstairs	ND^a^	ND	61.5%	69.8% (normal), 70.4% (brisk)
Downstairs	ND	ND	44.3%	79.4% (normal), 52.3% (brisk)

^a ^Not done.

**Table 7:** Methodology comparison with prior work.

Feature	Lu et al [10]	Reddy et al [11]	Kwapisz et al [12]	Current study
Device	Nokia N95 phone (iPhone unevaluated)	Nokia N95 phone	Android phones	iPhone/iPod Touch
Signal sources	Accelerometer	Accelerometer	Accelerometer, global positioning system	Accelerometer, gyroscope
Features	Mean, variance, mean crossing rate, spectrum peak, sub-band energy (ratio), spectral entropy	Mean, SD, average, absolute difference, magnitude, time between peaks, and binned distribution	Mean, variance, energy, and the density functional theory energy coefficients	Mean, SD, sum, magnitude, fast Fourier transform magnitude
Window sizes	4 seconds	1 second	10 seconds	2 seconds
Classifier	Support vector machine	Decision tree + Discrete Hidden Markov Model	Multilayer perception	k-nearest neighbor
Activities	Cycling, running, being still, riding in a vehicle, walking	Cycling, running, being still, riding in a vehicle, walking	Upstairs, downstairs, running, being still, walking	Upstairs (at different speeds), downstairs (at different speeds), running, being still, walking (at different speeds)

## Discussion

### Principal Results

This study is the first step in our effort to develop integrated tools to measure and intervene in physical activity and sedentary behavior. Combining time and frequency features of both acceleration and gyroscope measurements from sensors onboard smartphones, we classified common categories of physical activity and sedentary behavior (sitting, walking, and jogging at different paces) with high accuracy (90.1%–94.1%); up and down stair walking were classified at 52.3%–79.4% accuracies. Including orientation readings from a gyroscope proved to be beneficial for recognizing all activities studied.

### Limitation

We collected data using a convenience sample of participants. As motion pattern varies with individuals, future studies would benefit from using multiple demographic and physiological variables to inform participant designs. Furthermore, data were collected with the device placed in specific positions (armband for jogging, and shorts pocket for other activities). Jogging with only armband placement of the device likely influenced the signal pattern for this activity and may have contributed to the high classification accuracy we observed. Further investigation is needed to evaluate classification accuracies with more variable placement of the device (eg, hand, back pants pocket, or backpack). It will also be necessary to test the accuracy of activity classification in a free-living context, where individuals make natural transitions between activities such as sitting to standing and jogging to walking. Machine learning algorithms for classification often benefit from having diverse observations or subjects, because the machine can then learn more patterns of individual movement. Therefore, applying our classification methodology (features, window size, and classifiers) to a larger dataset would most likely result in higher accuracies.

We focused on classification of a somewhat narrow range of activities that pertained to ambulatory movements and sitting posture. Including other activities such as bicycling will be important to more fully capture the spectrum of physical activities in which people engage. However, classifying a wider range of activities might result in lower accuracies than were obtained in this study.

The nature of the false-positives shown in the confusion matrix was that when activities were misclassified it was usually by one speed gradation (eg, brisk walking misclassified as normal walking or jogging). This suggests that it might be possible to significantly improve accuracy by calibrating the prediction thresholds to individual users. This is an important area to explore in future work.

### Conclusion

This study is among the first to validate smartphone sensors including an accelerometer and gyroscope for activity recognition. The results suggest clear benefits of using a gyroscope as an additional data source for classifying activities. Including other signal data sources from the phone such as its global positioning system may further improve the system, but only for specifically identifying outdoor activities, and with the potential cost of reducing the battery life of the smartphone. Other sensors such as heart rate monitors might also further improve identifying the intensity of activities (eg, brisk walking compared with jogging). However, the trade-off of the extra burden of wearing an additional sensor would limit the public health impact of our system.

This study provided important indications of the possibilities and limitations of using a smartphone as an activity data collector. This system has potential high ecological validity because it requires people to carry only one device that they commonly carry with them already. The next step in our research is to test an onboard classifier application on the phone that can prompt users when needed for annotations in order to learn and classify individual activity patterns with high accuracy. The final step will be testing the feedback component that can offer individually tailored prompts and suggestions to increase physical activity and decrease sedentary time.

## Abbreviations

kNN: k-nearest neighbor
WEKA: Waikato Environment for Knowledge Analysis
